# An analytics-based heuristic decomposition of a bilevel multiple-follower cutting stock problem

**DOI:** 10.1007/s00291-021-00638-9

**Published:** 2021-05-28

**Authors:** Adejuyigbe O. Fajemisin, Laura Climent, Steven D. Prestwich

**Affiliations:** 1 Amsterdam Business School, University of Amsterdam, Amsterdam, The Netherlands; 2 Insight Centre for Data Analytics, School of Computer Science and IT, University College Cork, Cork, Ireland

**Keywords:** Analytics based, Bilevel, Cutting stock problem, Integer linear programming

## Abstract

This paper presents a new class of multiple-follower bilevel problems and a heuristic approach to solving them. In this new class of problems, the followers may be nonlinear, do not share constraints or variables, and are at most weakly constrained. This allows the leader variables to be partitioned among the followers. We show that current approaches for solving multiple-follower problems are unsuitable for our new class of problems and instead we propose a novel analytics-based heuristic decomposition approach. This approach uses Monte Carlo simulation and *k*-medoids clustering to reduce the bilevel problem to a single level, which can then be solved using integer programming techniques. The examples presented show that our approach produces better solutions and scales up better than the other approaches in the literature. Furthermore, for large problems, we combine our approach with the use of self-organising maps in place of *k*-medoids clustering, which significantly reduces the clustering times. Finally, we apply our approach to a real-life cutting stock problem. Here a forest harvesting problem is reformulated as a multiple-follower bilevel problem and solved using our approach.

## Introduction

Mathematical Optimisation has been used to solve practical problems in areas such as scheduling, planning, cutting stock, transportation as well as other fields (Vanderbei 2007; Kantorovich 1960; Bazaraa et al. 2010). In practical applications, however, optimisation problems are rarely straightforward since challenges such as several levels of optimisation and/or nonlinearities arise.

A bilevel optimisation problem is one in which one optimisation problem is nested within another. There is a leader (or outer/upper-level) problem, and a follower (or inner/lower-level) problem. The variables are split into leader variables and follower variables. The leaders solution must optimise an objective, with the constraint that the follower optimises a different objective. Both the leader and follower influence each other: the leader makes a decision while taking the followers objective into account, and the follower reacts to the leader’s decisions in an optimal manner. Due to this interaction, the solution of bilevel problems is difficult in the general case (Colson et al. 2007). Besides, bilevel problems become more difficult to solve when they contain nonlinearities (such as the problems analysed in this paper). A general mathematical formulation of the bilevel problems is as follows:

1 $$\begin{aligned} \begin{array}{r@{}l} \min _{\mathbf {x},\mathbf {y}} &{} F(\mathbf {x},\mathbf {y})\\ \text{ s.t. } &{} \mathbf {y} \in \text{ argmin}_{\mathbf {y}} \{f(\mathbf {x},\mathbf {y}) \,|\, g(\mathbf {x},\mathbf {y}) \le 0 \}\\ &{} G(\mathbf {x},\mathbf {y}) \le 0 \\ &{} \mathbf {x} \in X,\; \mathbf {y} \in Y \end{array} \end{aligned}$$

where $\mathbf {x}$ represents the leader decision vector and $\mathbf {y}$ the follower decision vector. The objective functions at the upper- and lower-level are represented by *F* and *f*, respectively. *G* and *g* represent the inequality constraints at the upper- and lower-levels, respectively, and equality constraints may also exist. *X* and *Y* are the bound constraints for the upper-level decision vector and lower-level decision vector, respectively. The condition $\mathbf {y} \in \text{ argmin}_{\mathbf {y}}$ forces $\mathbf {y}$ to be an optimal solution to the follower problem, and can be considered as a computationally expensive constraint.

One type of bilevel problem is the single-leader, multiple-follower problem, which has been used in applications like toll-setting, resource management, conflict resolution and many others. While several classical and evolutionary solution approaches exist for solving these multiple-follower problems, they are either not applicable in cases in which the follower problems are not traditional optimisation problems, or do not scale up appropriately. For this reason, in this paper, we present an alternative heuristic decomposition approach that decomposes the bilevel lower-level problem into smaller sub-problems (each sub-problem is composed of a follower) which are solved to optimality. Then, these solutions are used for computing an approximate solution to the upper-level bilevel problem (composed by the leader).

Furthermore, intending to reduce the computation time of solving this type of bilevel problems (which typically are large-scale when associated with real applications), our approach uses data-driven/analytics-based methods (specifically, we use clustering techniques). In data-driven optimisation, the goal is to use insights from any available data to improve the quality of the decisions made or the solution approaches taken. Analytics techniques can therefore be used to enhance decision making by learning useful features from the data or using data to simplify mathematical models, thus reducing the complexity and/or solution times of the models.

This paper is an extension of the work originally presented in the 2019 World Congress on Global Optimization (WCGO) Fajemisin et al. (2020). In this extension, we have focussed on providing a detailed literature review, the use of self-organising maps as an alternative to clustering and applying our approach to a real-life cutting stock problem: the forest harvesting problem. The main contributions of the work presented are: (i) a new class of multiple-follower bilevel problems is proposed; (ii) a novel analytics-based heuristic decomposition approach for solving this class of large-scale bilevel multiple-follower problems is given; (iii) the forest harvesting problem is reformulated as a bilevel optimisation problem to take into account the cutting operations of the harvesters.

This paper is organised as follows: a literature review of bilevel decomposition approaches is given in Sect. 2. The new class of bilevel multiple-follower problems we consider is given in Sect. 3. The heuristic decomposition method is described in Sect. 4, while numerical examples demonstrating the advantage of the analytics-based approach over evolutionary approaches are given in Sect. 5. Our approach is applied to the bilevel cutting stock problem in Sect. 6, and general conclusions are given in Sect. 7.

## Related work

Heuristic decomposition approaches divide the problem into subproblems, which are solved individually, and the results assembled into a solution to the original problem. None of these steps needs to be optimal, for example in Elkamel et al. (1997) the subproblems are solved optimally then combined into a near-optimal solution, while in Krüger et al. (1995) the subproblems are solved approximately. In these papers, scheduling problems are addressed. As an example of another application domain, in Lin et al. (2011) the authors present a decomposition heuristic for a network design problem. Our approach decomposes the problem into follower subproblems, and it is an approximate approach because we use sampling and clustering.

The following subsections provide an overview of the current solution approaches for bilevel problems and the motivation for an analytics-based approach.

### Decomposition approaches in the literature

Bilevel problems are known to be non-convex, non-differentiable and strongly $\mathcal {NP}$-hard even in the simplest cases (Colson et al. 2007), and so most approaches for solving them involve some form of decomposition of the problem into more tractable forms. The most common method for single-level reduction is to replace the lower-level problem with its Karush–Kuhn–Tucker (KKT) (Bertsekas 1999) conditions (Bard 1984; Dempe and Zemkoho 2012). The authors in Visweswaran et al. (1996) present a decomposition approach for solving linear and quadratic bilevel problems. They transform the problem into a single-level one by replacing the inner problem with its KKT conditions. The problem is then decomposed into a series of primal and relaxed-dual sub-problems, whose solutions are used as lower and upper bounds. This procedure is run iteratively until a global optimum is found. This method is similar to that in Zeng and An (2014), where the problem is first reformulated into a single-level problem using KKT conditions and strong duality. Then, a similar iterative process is carried out until an optimal solution is found.

More complex approaches combine the use of the KKT conditions with other techniques. For example, in Kristianto et al. (2013) the stochastic bilevel problem is reduced to a single level using KKT conditions and is then solved using Benders’ decomposition. Related approaches include (Saharidis and Ierapetritou 2009), where an algorithm for solving mixed-integer bilevel linear problems based on Benders’ decomposition is presented. Similarly, (Raidl et al. 2014) uses logic-based Benders’ decomposition to solve a bilevel vehicle routing problem, which is combined with a variable neighbourhood search heuristic to speed up search time and improve scalability. To address the problem of weak Benders’ cuts, (Nishi et al. 2011) uses a Lagrangian relaxation method to generate stronger cuts for simultaneous scheduling and routing problem for automated guided vehicles.

In Dempe and Franke (2016) lower-level problems are replaced with their Fritz-John conditions (John 1948), and an algorithm is presented for solving problems with fully convex lower-levels. This method is applied in Dempe and Franke (2014) to solve a bilevel road pricing problem. Nogales Martín and Miguel (2004) show a relationship between one bilevel decomposition algorithm and a direct interior-point method based on Newton’s method. The authors in Iyer and Grossmann (1998) present a decomposition algorithm for solving a network planning problem. The upper level is solved to get an upper bound, which is then used to get a solution for the lower level problem which provides a lower bound. The process occurs iteratively, adding integer cuts along the way until a small enough gap between the bounds is achieved.

In Sugiyama et al. (2012), the authors solve a railway crew rostering problem. Their decomposition is in the form of cuts to reduce the feasible region of the master problem. Local search is also incorporated to improve the upper bound generated by solving the sub-problems. In Caramia and Mari (2016) the decomposition algorithm proposed consists of solving the single-level relaxation (SLR) of the Bilevel Facility Location (BFL) problem, solving the slave problem (SVP) which is the BFL for a given fixed set of open facilities, generating cuts based on the structure of the problem, and repeating until a stopping criterion is reached.

As with single-follower bilevel problems, both classical and evolutionary approaches have been used for solving Bilevel Multiple-Follower (BLMF) problems. Lu et al. (2006) presents a general framework and solutions for these problems. Nine classes of multiple-follower problems are presented (none of which include the problem class proposed in Sect. 3) with corresponding models presented for each class. Also, an extended Kuhn-Tucker approach is presented for solving the uncooperative model to optimality. A practical example in the form of a road network problem is given. Similarly, (Lu et al. 2005) uses a Kuhn-Tucker (KT) approach for BLMF problems in which the followers may or may not have shared variables. Shi et al. (2005) and Lu et al. (2007) use extended KT approaches. In Lu et al. (2007) a branch-and-bound algorithm is used to solve the problems with referential-uncooperative followers. Calvete and Galé (2007) reformulates a problem with multiple followers into one with one leader and one follower by replacing the lower levels with an equivalent objective and constraint region. This method cannot be applied to the BPMSIF, as neither its objectives nor its inducible region are equivalent to those of the problem class addressed in Calvete and Galé (2007).

There is also literature on applying the *K*th-best approach to problems with multiple followers. Shi et al. (2005) presents the theoretical properties of BLMFs and *K*th-best approach for solving such problems, while (Shi et al. 2007) uses the approach for problems with shared variables among followers. Similarly, (Zhang et al. 2008b) presents an extended *K*th-best approach for solving referential-uncooperative BLMF decision problems, and provides an application in the form of a logistics planning problem.

Fuzzy approaches to solving BLMFs include (Wang et al. 2009) which uses a fuzzy interactive algorithm to solve problems with partially shared variables among the followers. Zhang and Lu (2010) combines fuzzy models and a *K*th-best algorithm to solve cooperative multiple-follower problems. Fuzzy models combined with a branch-and-bound algorithm have also been used in Zhang et al. (2008a, b, 2007) to solve problems with shared decision variables among the followers.

Decomposition approaches that also incorporate evolutionary approaches exist. A Co-Evolutionary Decomposition-based Bilevel Algorithm (CODBA) is presented in Chaabani et al. (2015), in which an algorithm is first used to generate a set of points from a discrete solution space. This allows them to generate a population of solutions for the lower-level problem. Several sub-populations of the lower-level problem are generated, and the best individuals in the sub-populations are allowed to co-evolve. CODBA II (Chaabani et al. 2015) is an improvement in which parallelism and co-evolution are implemented at both levels of the bilevel problem. Evolutionary approaches also exist where an iterative approximation of the reaction set is used to approximate the lower-level problem (Sinha et al. 2013, 2017, 2014). Additionally, decomposition approaches involving evolutionary approaches are given in Li and Wang (2010); Li et al. (2016). In terms of multiple-follower problems, the literature includes (Angelo and Barbosa 2015), where a differential evolution method is used to solve cases in which there is information shared between the followers. Liu (1998) presents a genetic algorithm for solving nonlinear multilevel problems with multiple followers. Also, (Islam et al. 2016) extend their bilevel memetic algorithm to solve problems with multiple followers using a combination of global and local search. The authors in Ke et al. (2016) combine fuzzy programming with an evolutionary algorithm, as well as neural networks to solve a multi-follower problem with non-cooperative followers. Reviews on the use of metaheuristic approaches in bilevel optimisation are given in Talbi (2013); Sinha et al. (2017).

### Motivation for the analytics-based heuristic decomposition approach

In summary, both classical and evolutionary approaches have been applied to single- and multi-follower problems. Lu et al. (2006) presents a general framework and solutions for nine classes of multi-follower problem, but none are applicable to the new class of problems we consider in Sect. 3. The authors in Calvete and Galé (2007) reformulate a problem with multiple followers into one with one leader and one follower, by replacing the lower levels with an equivalent objective and constraint region. This method also cannot be applied to our problem, as neither its objectives nor its inducible region are equivalent to the problem class of Calvete and Galé (2007). Additionally, the methods proposed in Calvete and Galé (2007); Lu et al. (2006) assume that the followers are linear, which is not the case with our class of problems. Most classical methods for handling bilevel problems require assumptions of smoothness, linearity or convexity, while we make no such assumptions. Evolutionary and meta-heuristic techniques also do not make these assumptions (Angelo and Barbosa 2015; Islam et al. 2016; Liu 1998) but most are computationally intensive nested strategies. They are efficient for smaller problems but do not scale up well to large-scale problems. In contrast, our analytics-based approach scales up well as the number of followers increases (see Sect. 5).

## A new class of bilevel problems

In Bilevel Multi-Follower (BLMF) problems there may be several followers, and multi-leader problems are also known.

For a BLMF problem with *Q* followers, let $\mathbf {x}$ represent the leader decision vector, and $\mathbf {y}_q$ the decision vector for follower *q* ($q=1 \ldots Q$). The leader chooses a strategy $\mathbf {x}$, following which each follower selects its own strategy in response to $\mathbf {x}$. The BLMF problem may be either cooperative, partially cooperative or uncooperative, depending on the leader and follower objectives. Based on the type of interaction between followers, nine classes of linear BLMF problems are identified in Lu et al. (2006). Problems in which the followers do not share objectives or constraints are known as “independent” and take the form:

$$\begin{aligned} \begin{array}{ll} \min _{\mathbf {x}, \mathbf {y}_1 \ldots \mathbf {y}_Q} \, &{} F(\mathbf {x},\mathbf {y}_1, \ldots , \mathbf {y}_Q)\\ \text{ s.t. } &{} G(\mathbf {x}, \mathbf {y}_1, \ldots , \mathbf {y}_Q) \le 0\\ &{} \mathbf {x}_q \in X_q,\\ &{} \text{ where } \text{ each } \mathbf {y}_q \, (q = 1, \ldots , Q) \text{ solves }\\ &{} \min _{\mathbf {y}_q} \, f(\mathbf {x},\mathbf {y}_1, \ldots , \mathbf {y}_Q)\\ &{} \text{ s.t. } g(\mathbf {x},\mathbf {y}_1, \ldots , \mathbf {y}_Q) \le 0 \end{array} \end{aligned}$$

Several researchers have worked on bilevel optimisation with *multiple independent followers* (Lu et al. 2006; Zhang and Lu 2010). However, we strengthen this independence condition to one we call *strong independence*.

### **Definition 1**

A Bilevel Problem with Multiple Strongly Independent Followers (BPMSIF) is one in which:

(i) the followers do not share each others’ follower *or leader* variables, so that $\mathbf {x}$ can be partitioned into *q* parts: $\mathbf {x}_q$ ($q=1 \ldots Q$);
(ii) follower functions $f_q(\mathbf {x}_q,\mathbf {y}_q)$ are allowed to be nonlinear;
(iii) variables from different follower problems are not tightly mutually constrained.

In (iii) weak constraints such as a single linear inequality are allowed. Thus the BPMSIF has the form:

2 $$\begin{aligned} \begin{array}{r@{}l@{}l} \min _{\mathbf {x}_1 \ldots \mathbf {x}_Q, \mathbf {y}_1 \ldots \mathbf {y}_Q} &{} F(\mathbf {x}_1 , \ldots , \mathbf {x}_Q,\mathbf {y}_1 , \ldots , \mathbf {y}_Q) &{}\\ \text{ s.t. } &{} G(\mathbf {x}_1 , \ldots , \mathbf {x}_Q,\; \mathbf {y}_1 , \ldots , \mathbf {y}_Q) \le 0 \\ &{} \text{ where } \text{ each } \mathbf {y}_q \, (q = 1, \ldots , Q) \text{ solves } \\ &{} \min _{\mathbf {y}_q} \, f_q(\mathbf {x}_q,\mathbf {y}_q) \\ &{} \text{ s.t. } g_q(\mathbf {x}_q,\mathbf {y}_q) \le 0 \\ &{} \mathbf {y}_q \in Y_q \end{array} \end{aligned}$$

where $F,f_q$ may be any (possibly nonlinear) objective functions, $G,g_q$ may be any set of (possibly nonlinear) constraints, the *G* constraints are weak, and $X_q,Y_q$ may be vectors of any variable domains (real, integer, binary, or richer Constraint Programming domains such as set variables). Examples of bilevel real applications with nonlinearities are (Herskovits et al. 2000), (John 1948) and (Calvete et al. 2011). The last two are applied to planning problems. In this paper, in Sect. 6 we address a cutting stock problem associated with a forestry harvesting real application, which is a BPMSIF, as stated in Definition 1.

Problem (2) satisfies the features of a BPMSIF. Firstly, each follower function here can be seen to be a function of only its variables $\mathbf {y}_q$ and a portion of the leader’s variables $\mathbf {x}_q$. Secondly, $G(\mathbf {x}_1 , \ldots , \mathbf {x}_Q,\; \mathbf {y}_1 , \ldots , \mathbf {y}_Q) \le 0$ is weak and may, for example, take the form of a simple weighted sum such as $\sum _q^Q B_q y_q \le b$, where the $B_q$ and *b* are constants. The BPMSIF is different from multi-leader problems such as those of (DeMiguel and Xu 2009; Lu et al. 2016; Ramos et al. 2016). Its constraint region is:

$$\begin{aligned} \Omega = \{ (\mathbf {x}_1, \ldots , \mathbf {x}_Q,&\mathbf {y}_1, \ldots , \mathbf {y}_Q) \in \, X_1 \ldots \times X_Q \times Y_1 \times \ldots \times Y_Q : \nonumber \\&G(\mathbf {x}_1, \ldots , \mathbf {x}_Q,\mathbf {y}_1, \ldots , \mathbf {y}_Q) \le 0, g(\mathbf {x}_q,\mathbf {y}_q) \le 0, \, q = 1, \ldots , Q \} \end{aligned}$$

The projection of $\Omega$ onto the leader’s decision space is:

$$\begin{aligned} \Omega (\mathbf {x}) = \{ \mathbf {x}_q \in X_q : \,&\exists \mathbf {y}_q \in Y_q : G(\mathbf {x}_1, \ldots , \mathbf {x}_Q,\mathbf {y}_1, \ldots , \mathbf {y}_Q) \le 0, \nonumber \\&g(\mathbf {x}_q,\mathbf {y}_q) \le 0, \, q = 1, \ldots , Q \} \end{aligned}$$

The feasible set for follower *q* is affected by a corresponding part $\mathbf {x}_q$ of a given leader decision vector so that:

$$\begin{aligned} \Omega _q (\mathbf {x}_q) = \{ \mathbf {y}_q : (\mathbf {x}_q, \mathbf {y}_q) \in \Omega \} \end{aligned}$$

Each follower’s rational reaction set is given as:

$$\begin{aligned} \Psi _q (\mathbf {x}_q) = \{ \mathbf {y}_q \in Y_q: \mathbf {y}_q \in \text{ argmin } f_q(\mathbf {x}_q,\mathbf {y}_q) \, | \, \mathbf {y}_q \in \Omega _q (\mathbf {x}_q) \} \end{aligned}$$

Finally, the inducible region ($\mathcal {IR}$) is:

$$\begin{aligned} \mathcal {IR} = \{ (\mathbf {x}_1, \ldots , \mathbf {x}_Q, \mathbf {y}_1, \ldots , \mathbf {y}_q):&(\mathbf {x}_1, \ldots , \mathbf {x}_Q, \mathbf {y}_1, \ldots , \mathbf {y}_q) \nonumber \\&\in \Omega , \, \mathbf {y}_q \in \Psi _q (\mathbf {x}), \, q = 1, \ldots , Q \} \end{aligned}$$

As in standard bilevel programming *min* and *argmin* have been used without loss of generality: each subproblem may involve maximisation.

## Analytics-based heuristic decomposition approach

In this section, we first explain the Monte Carlo simulation and then we analyse two alternatives: *k*-medoids clustering and self-organising maps.

### Monte Carlo simulation

For each follower *q* a large number *S* of feasible solutions for the leader vector $\mathbf {x}_q$ associated with that follower are generated, using Monte Carlo simulation. To avoid bias the $\mathbf {x}_q$ are generated using Hypersphere Point Picking (Marsaglia 1972; Muller 1959), which uniformly samples from a vector space. This results in a set $\mathbf {X}_{sq}$ ($s=1 \ldots S$, $q=1 \ldots Q$). The associated follower problems are then solved using the $\mathbf {X}_{sq}$ to obtain a corresponding set of follower vectors $\mathbf {Y}_{sq}$. We now have multiple potential leader solutions, together with their corresponding follower solutions for each follower problem $f_q(\mathbf {x}_q,\mathbf {y}_q)$.

### *k*-medoids clustering

In order to model and solve the BPMSIF as an Integer Linear Program (ILP), the large number of potential solutions $\mathbf {X}_{sq}$ must be reduced to a manageable size, which we do via *k*-medoids clustering (de Amorim and Fenner 2012). Unlike *k*-means where centroids are means of data points, *k*-medoids selects actual data points. Once the large set $\mathbf {X}_{sq}$ has been clustered using *K* clusters, the medoids from each cluster are selected to represent the large generated set. The corresponding $\mathbf {Y}_{kq}$ are then selected from $\mathbf {Y}_{sq}$ so that we now have a smaller representative set of assignments $\mathbf {X}_{kq}$ and $\mathbf {Y}_{kq}$. The most common algorithm for *k*-medoid clustering is the Partitioning Around Medoids (PAM) algorithm (de Amorim and Fenner 2012). This is inefficient on very large datasets, so instead we use the CLARA algorithm which is a combination of PAM and random sampling (Kaufman and Rousseeuw 2009; Wei et al. 2000). The BPMSIF can now be transformed into a single-level optimisation problem:

$$\begin{aligned} \begin{array}{r@{}l@{}l} \min _{\mathbf {x}_1 \ldots \mathbf {x}_Q} &{} F(\mathbf {x}_1 \ldots \mathbf {x}_Q,\mathbf {y}_1 \ldots \mathbf {y}_Q) &{}\\ \text{ s.t. } &{} G(\mathbf {x}_1 \ldots \mathbf {x}_Q,\, \mathbf {y}_1 \ldots \mathbf {y}_Q) \le 0&{}\\ &{} \mathbf {x}_q=\mathbf {X}_{kq} \rightarrow \mathbf {y}_q=\mathbf {Y}_{kq} &{} (q=1 \ldots Q,\; k=1 \ldots K)\\ &{} \mathbf {x}_q \in \{\mathbf {X}_{kq} \,|\, k=1 \ldots K\} &{} (q=1 \ldots Q)\\ &{} \mathbf {y}_q \in \{\mathbf {Y}_{kq} \,|\, k=1 \ldots K\} &{} (q=1 \ldots Q) \end{array} \end{aligned}$$

The constraint $\mathbf {x}_q=\mathbf {X}_{kq} \rightarrow \mathbf {y}_q=\mathbf {Y}_{kq}$ ensures that if $\mathbf {x}_q$ is assigned a value in $\mathbf {X}_{kq}$ then $\mathbf {y}_q$ is assigned the corresponding value in $\mathbf {Y}_{kq}$. This constraint can either be linearised using the big-M approach, or implemented directly using CPLEX indicator constraints (IBM 2017).

### Self-organising maps: an alternative to clustering

**Fig. 1:**
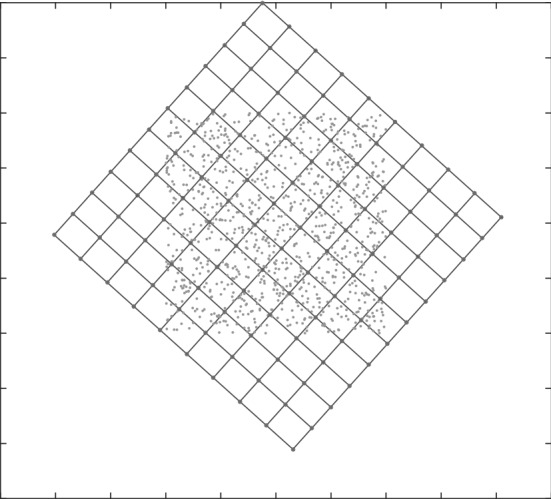
SOM lattice over data

**Fig. 2:**
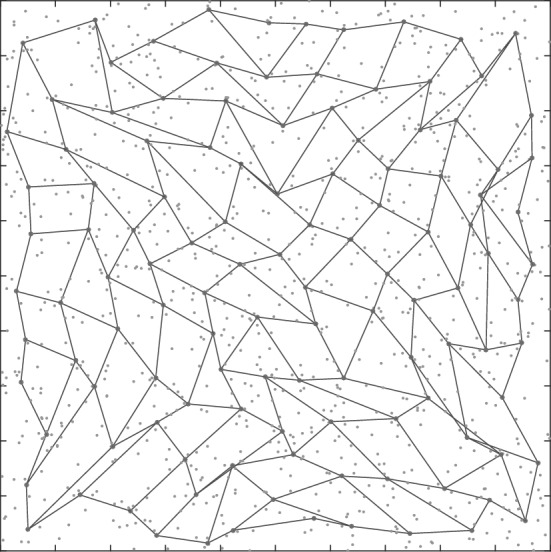
Map aligned with data

**Fig. 3:**
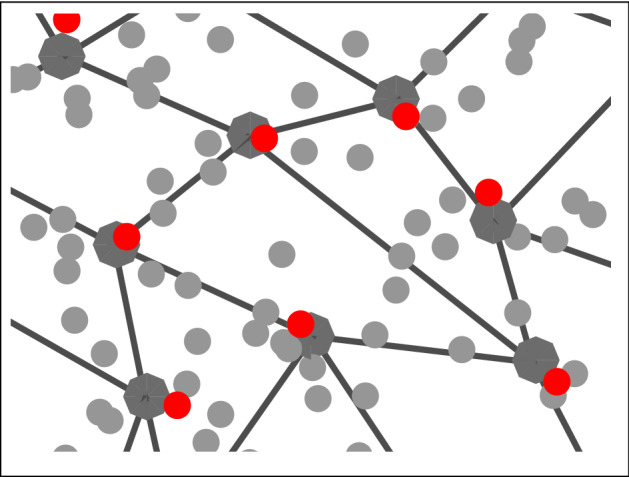
Selection of points closest to neurons

As an alternative to *k*-medoids clustering (which can be time consuming), a neural network approach is also considered. Self-organising maps (SOMs) are a type of artificial neural network in which, given a set of input data vectors, individual neurons in the map compete to align themselves with the vectors they are best matched with (Kohonen 1982). SOMs operate in two phases—a training phase in which the map is built using sample data, and a mapping phase in which new data is automatically classified based on the map built in the previous phase, with the number of neurons of the SOM determining the number of classes. In this paper however, the intention is not to use SOMs for classifying new data. Instead, the map generated by the SOM trained over the large data set is used to select a smaller but highly representative subset of the data. (Note that the size of the reduced set is equal to the number of neurons.) This is analogous to clustering (Bullinaria 2004; Lampinen and Oja 1992). This reduced dataset is then used to solve the bilevel optimisation problem. Figures 1, 2 and 3 give an illustration of this approach.

Starting with a 2-D lattice of neurons, as shown in Fig. 1, the map is trained on the underlying data. After several iterations, the map will have aligned itself with the underlying distribution, as shown in Fig. 2. Once the map is aligned, the data points closest to each neuron is selected, one data point for each neuron. Figure 3 shows a zoomed-in section of Fig. 2, with the neurons as octagonal structures and the selected data points in red. This reduced dataset can then be used to solve the transformed multiple-follower bilevel problem.

To use the SOM for clustering, i.e. reducing $\mathbf {Y}_{sq}$ to $\mathbf {Y}_{kq}$, the number of neurons in the lattice is first set to *K*. The SOM is then trained on the $\mathbf {Y}_{sq}$ over several iterations. Once the training phase is complete, the closest follower vector to each neuron is selected, resulting in a set $\mathbf {Y}_{kq}$. Finally, the leader vectors $\mathbf {X}_{kq}$ corresponding to the $\mathbf {Y}_{kq}$ are selected, resulting in a clustered set.

## Numerical examples

To illustrate and evaluate our approach, we use two example problems. Monte Carlo simulation and clustering were done in Java and R (using the CLARA package Maechler et al. (2017)), respectively. The CPLEX 12.6 solver was also used on a 3.0 GHz Intel Xeon Processor with 8 GB of RAM.

### A benchmark problem

The first problem considered, a two-follower problem, is Example 2 from (Bard 1988), and is used simply to illustrate our approach. This toy problem can be solved optimally with complete methods, but it is given here simply to demonstrate our technique (which is meant to be used in much larger problems).

3 $$\begin{aligned} \begin{array}{r@{}l@{}l@{}l} \max &{} F(\mathbf {x},\mathbf {y}_1,\mathbf {y}_2) = (200-y_{11}-y_{21})(y_{11}+y_{21}) \\ &{} +(160-y_{12}-y_{22})(y_{12}+y_{22}) &{}\\ \text{ s.t. } &{} x_1 + x_2 + x_3 + x_4 \le 40\\ &{} 0 \le x_1 \le 10, 0 \le x_2 \le 5, 0 \le x_3 \le 15, 0 \le x_4 \le 20\\ &{} \\ &{} \min f_1(\mathbf {y}_1) = (y_{11}-4)^2 + (y_{12} -13)^2 \;\text{ s.t. } \\ &{} 0.4y_{11} + 0.7y_{12} \le x_1, \;0.6y_{11} + 0.3y_{12} \le x_2, \;0 \le y_{11} , y_{12} \le 20 \\ &{} \\ &{} \min f_2(\mathbf {y}_2) = (y_{21}-35)^2 + (y_{22}-2)^2 \;\text{ s.t. } \\ &{} 0.4y_{21} + 0.7y_{22} \le x_3, 0.6y_{21} + 0.3y_{22} \le x_4, 0 \le y_{21} , y_{22} \le 40 \end{array} \end{aligned}$$

This is a BPMSIF as the followers are strongly independent: the followers do not share each others’ follower or leader variables, and the follower problem variables are not mutually constrained. The leader vector $\mathbf {x} = (x_1, x_2, x_3, x_4)$ is partitioned among the followers with variables $(x_1, x_2)$ occurring in follower 1 and $(x_3, x_4)$ in follower 2. The variables $\mathbf {y_1} = (y_{11},y_{12})$ and $\mathbf {y_2} = (y_{21},y_{22})$ are also computed by followers 1 and 2, respectively.

To solve this problem using the analytics-based decomposition method, denote $(x_1, x_2)$ by a vector $\mathbf {\lambda }_1$ and $(x_3, x_4)$ by a vector $\mathbf {\lambda }_2$. A large number *S* of assignments for $\mathbf {\lambda }_1$ and $\mathbf {\lambda }_2$ which satisfy the bounds of the $\mathbf {x}$’s are generated (Marsaglia 1972; Muller 1959), and denoted by $\mathbf {\Lambda }_{s1}$ and $\mathbf {\Lambda }_{s2}$ ($s = 1 \ldots S$), respectively. For each $\mathbf {\lambda }_1$ in $\mathbf {\Lambda }_{s1}$ the corresponding follower problem $\mathbf {f_1}$ is solved as an ILP, obtaining assignments $\mathbf {Y}_{s1}$; similarly for $\mathbf {Y}_{s2}$. Next, the $\mathbf {Y}_{s1}$ vectors are clustered using *k*-medoids to get the most diverse set of assignments $\mathbf {Y}_{k1}$, ($k = 1 \ldots K$). The $\mathbf {\Lambda }_{k1}$ vectors that correspond to the $\mathbf {Y}_{k1}$ are then selected. The same is done for $\mathbf {Y}_{s2}$ to obtain $\mathbf {Y}_{k2}$ along with its corresponding $\mathbf {\Lambda }_{k2}$. Using this decomposition, problem (3) can now be rewritten as a standard optimisation problem:

4 $$\begin{aligned} \begin{array}{r@{}l@{}l} \max &{} \mathbf {F}(\mathbf {x},\mathbf {y}_1 \ldots \mathbf {y}_2) = (200-y_{11}-y_{21})(y_{11}+y_{21}) \\ &{} +(160-y_{12}-y_{22})(y_{12}+y_{22}) &{}\\ \text{ s.t. } &{} \lambda _{11} + \lambda _{12} + \lambda _{21} + \lambda _{22} \le 40 &{}\\ &{} u_k = 1 \rightarrow \mathbf {\lambda }_1 = \mathbf {\Lambda } _{k1},\; u_k = 1 \rightarrow \mathbf {y}_1 = \mathbf {Y} _{k1} &{} k = 1 \ldots K\\ &{} v_k = 1 \rightarrow \mathbf {\lambda }_2 = \mathbf {\Lambda } _{k2},\; v_k = 1 \rightarrow \mathbf {y}_2 = \mathbf {Y} _{k2} &{} k = 1 \ldots K\\ &{} \sum _k^K u_k = 1,\; \sum _k^K v_k = 1 \end{array} \end{aligned}$$

where $\lambda _{11} = x_1$, $\lambda _{12} = x_2$, $\lambda _{21} = x_3$ and $\lambda _{22} = x_4$. This model can be linearised using the big-M approach, but the ILP is solved faster when CPLEX indicator constraints are used.^1^ The binary variables $u_k$ and $v_k$ ensure that only one assignment each is selected from $\mathbf {\Lambda }_{k1}$ and $\mathbf {Y}_{k1}$, and from $\mathbf {\Lambda }_{k2}$ and $\mathbf {Y}_{k2}$, respectively. The $\lambda _{11} + \lambda _{12} + \lambda _{21} + \lambda _{22} \le 40$ constraint ensures that an $(x_1, x_2)$ and an $(x_3, x_4)$ that satisfy the original constraints on the $\mathbf {x}$ are selected.

In experiments, as *K* increases better solutions were found, with the highest value of 6594.05 obtained when $K=160$ giving $\mathbf {x} = (8.13,3.80,11.23,16.82)$, $\mathbf {y}_1 = (0.74,11.20)$ and $\mathbf {y}_2 = (28.04,0.00)$ (rounded to 2 decimal places). The clustering time when $K=160$ is 234.53 s. The solution is $0.09\%$ less than optimal, but the strength of our approach is in its ability to handle large-scale problems, as demonstrated next.

### A large-scale problem

In this experiment, a problem with arbitrarily many followers is evaluated. The problem is also evaluated for the optimistic case in which the followers’ solutions lead to the best objective function value for the leader.

5 $$\begin{aligned} \begin{array}{r@{}l@{}l} \max &{} \sum _q^Q \mathbf {a}_q \mathbf {x}_q + \sum _q^Q \mathbf {b}_q \mathbf {y}_q &{}\\ \text{ s.t. } &{} \mathbf {x}_q \in {\mathbb {R}}^N &{} q=1 \ldots Q\\ &{} x_{qn} \le x_{qn}^{max} &{} q=1 \ldots Q,\; n=1 \ldots N\\ &{} \mathbf {y}_q \in \text{ argmin } \mathbf {c}_q \mathbf {x}_q + \mathbf {d}_q \mathbf {y}_q &{} q=1 \ldots Q\\ &{} \text{ s.t. } \;\sum _n^N y_{qn} \le \sum _n^N x_{qn} &{} q=1 \ldots Q\\ &{} e_{qn} x_{qn} \le y_{qn} \le y_{qn}^{max} &{} q=1 \ldots Q,\; n=1 \ldots N \end{array} \end{aligned}$$

where $\sum _q \mathbf {a}_q \mathbf {x}_q = \sum _q \sum _n a_{qn} x_{qn}$, $\mathbf {x}$ and $\mathbf {y}$ are the variables controlled by the leader and followers, respectively, and *Q* is the total number of followers. Both the $\mathbf {x}$ and $\mathbf {y}$ are vectors of real numbers. The leader variables are partitioned among the followers such that each follower contains one $\mathbf {x}_q$ each, and each $\mathbf {x}_q$ is of size *n*. Each component of the vector $x_{qn}$ is constrained to be $\le$ a given upper bound $x_{qn}^{max}$. $\mathbf {a}_q$, $\mathbf {b}_q$, $\mathbf {c}_q$, $\mathbf {d}_q$ and $\mathbf {e}_q$ are vectors of constants.

The decomposition approach outlined in Sect. 4 was used to decompose the problem, which is then written as:

$$\begin{aligned} \begin{array}{r@{}l@{}l} \max &{} \sum _q^Q \sum _n^N a_{qn} x_{qn} + \sum _q^Q \sum _n^N b_{qn} y_{qn} &{}\\ \text{ s.t. } &{} x_{qn} - X_{kqn} \le M(1-u_{kq}) &{} k = 1 \ldots K,\; q = 1 \ldots Q, n = 1 \ldots N\\ &{} X_{kqn} - x_{qn} \le M(1-u_{kq}) &{} k = 1 \ldots K,\; q = 1 \ldots Q,\; n = 1 \ldots N\\ &{} y_{qn} - Y_{kqn} \le M(1-u_{kq}) &{} k = 1 \ldots K,\; q = 1 \ldots Q,\; n = 1 \ldots N\\ &{} Y_{kqn} - y_{qn} \le M(1-u_{kq}) &{} k = 1 \ldots K,\; q = 1 \ldots Q,\; n = 1 \ldots N\\ &{} \sum _k^K u_{kq} = 1 &{} q = 1 \ldots Q\\ &{} u_{kq} \in \{0,1\} &{} k = 1 \ldots K,\; q = 1 \ldots Q \end{array} \end{aligned}$$

where *M* is a sufficiently large constant.

#### Evaluation

**Fig. 4:**
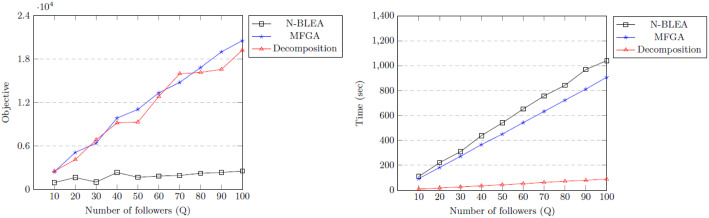
Comparing approaches: objectives and timings for $Q = 10 \text{ to } 100$

**Fig. 5:**
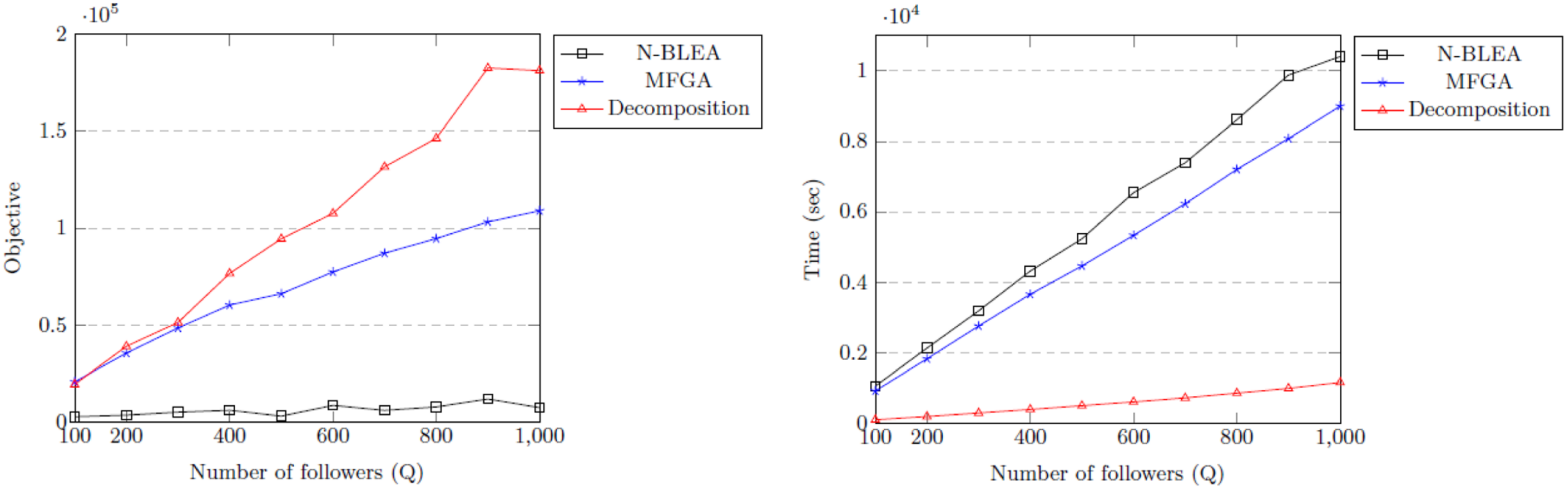
Comparing approaches: objectives and timings for $Q = 100 \text{ to } 1000$

The values used for the problem are $$N = 6$$, $$x_{qn}^{min} = 0$$, $$x_{qn}^{max} = 10$$, $$y_{qn}^{max} = 10$$, ($$\forall q,n$$). $$a_{qn}$$, $$b_{qn}$$, $$c_{qn}$$, and $$d_{qn}$$ are Gaussian random real variables in [0.0, 15.0), [0.0, 20.0), $$[-10.0, 10.0)$$ and $$[-12.0, 12.0)$$, respectively. $$e_{qn}$$ is a uniform random real variable in [0.0, 1.0). The number of followers *Q* was varied between 10 and 1000, and the problem was solved using both our analytics-based heuristic decomposition approach (using $$S=1000$$, $$K = 30$$ for each follower) and two genetic algorithms, and the results are shown in Figs. 4 and 5. Genetic algorithms are frequently used in bilevel optimisation, so this evaluation looks at the performance of this approach for the example problem. The first genetic algorithm is the Nested Bilevel Evolutionary Algorithm (N-BLEA) used in Sinha et al. (2014) and has been well-used for solving bilevel problems. The second is the Multi-Follower Genetic Algorithm (MFGA) described in Algorithm 1 and specifically designed for this problem.

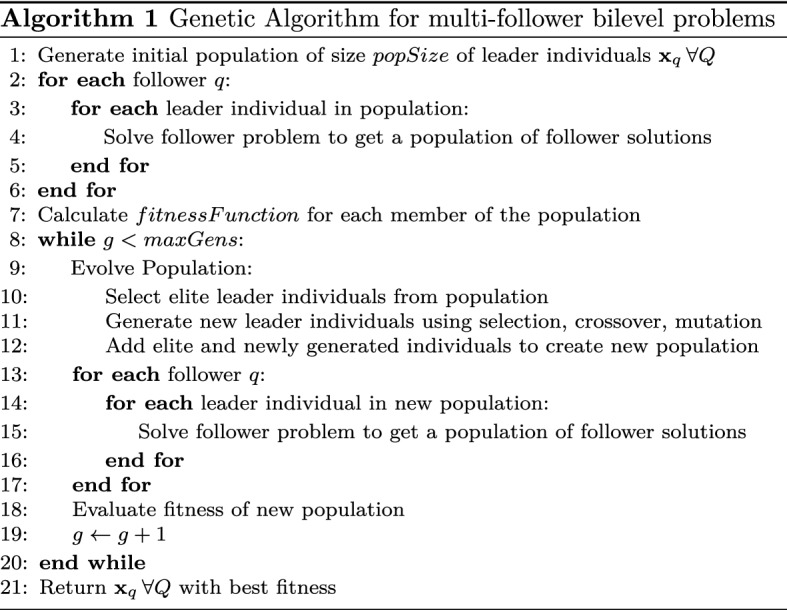



#### N-BLEA parameters

In order to select the parameters to use, the problem with 100 followers ($Q = 100$) was first solved while varying some algorithm parameters. The number of parents $\mu$ and number of offspring $\lambda$ ($\mu = \lambda$) were varied from 3–8. For each of these values, the number of generations (*maxGens*) was also varied from 50–200 in steps of 50. This operation was run 10 times for each value of $\mu$, $\lambda$ and *maxGens*, and the average objective value was recorded.

It was seen that the following settings produced the best solutions on average: $\mu = \lambda = 8$, number of generations $maxGens = 150$, $\text{ tournamentSize } = 5$, number of random individuals added to pool $r = 2$, $\text{ crossoverProbability } = 0.9$ and $\text{ mutationProbability } = 0.1$. The constraint handling method used by the algorithm is given in Deb (2000), and the variance-based termination criteria were set to 0.000001.

#### MFGA parameters

These were also varied using 100 followers. The population size $\text{ popSize }$ was varied from 30–90, and the maximum number of generations $\text{ maxGens }$ from 50–500. The MFGA parameters selected were therefore: $\text{ maxGens } = 500$, $\text{ popSize } = 50$. This population size was selected because, although there is little difference between its objective value and the best objective at $\text{ popSize }=80$, the difference in time taken is almost $50\%$ less. Uniform crossover with a crossover rate of 0.5 ($50\%$) was used. Other parameters are $\text{ elitePercentage } = 0.20$, $\text{ tournamentSize } = 5$, $\text{ mutationRate } = 0.015$ and $\text{ fitnessFunction } = \sum _q^Q \sum _n^N a_{qn} x_{qn} + \sum _q^Q \sum _n^N b_{qn} y_{qn}$.

#### Comparing all 3 approaches

For both N-BLEA and MFGA, each problem size was solved 10 times, and the average objective values and solution times were recorded. It should be noted that the poor performance of N-BLEA is due to the operation of its crossover operator which is additive in nature, and frequently violates the bounds of the vectors. This crossover operator results in offspring which are frequently infeasible, and are thus heavily penalised by the constraint handling scheme. MFGA was designed to avoid this problem: since vector generation is done using Hypersphere Point Picking with the appropriate boundaries, it always produces feasible offspring.

For 10–100 followers, the solution found by the MFGA was better in 7 out of 10 of the cases, though our approach finds a close solution in a fraction of the time (Fig. 4). However, as the problems get larger, (*Q* from 100 to 1000) our approach is much better in terms of both the solution quality and the runtime (Fig. 5), especially as *Q* gets larger. This demonstrates the scalability of our approach. Reduction in a very large set of potential solutions to a much smaller (but highly representative) set using medoids allows the ILP to choose the best solution from a vast number of possibilities.

## A large-scale bilevel cutting stock problem

In this section we first explain the real application of forestry harvesting. Then, we provide its bilevel reformulation and we evaluate it.

### A multiple stock size cutting stock problem

In the classic CSP, all stock items have the same known dimensions, which makes it easier to compute the possible cutting patterns. In certain problems however, stock items come in several different dimensions and these types of problems are known as Multiple Stock Size Cutting Stock Problems (MSSCSP) (Wäscher et al. 2007).

**Fig. 6:**
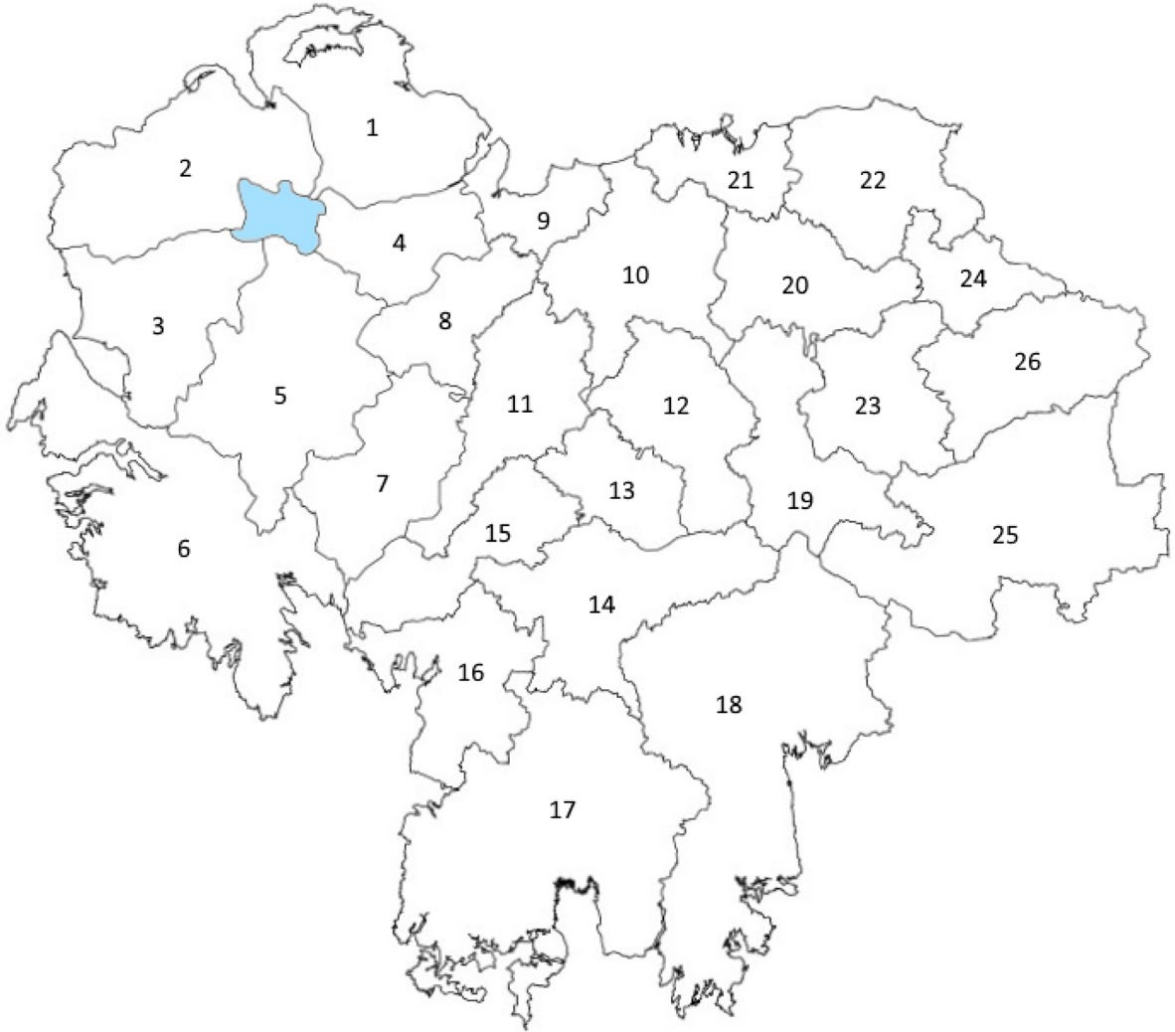
Schematic view of a forest partitioned into blocks

An example of such a problem is the forest harvesting problem, as the trees differ in size from each other, sometimes significantly. In this problem, a forest is subdivided into areas called “blocks”, with each block having a number of trees to harvest. This partitioning is illustrated in Fig. 6.

There are *Q* blocks each with market value $V_q$ ($q=1 \ldots Q$). Each block has *R* trees, with tree *r* ($r=1 \ldots R$) in block *q* having dimensions $\mathbf {\sigma }_{qr}$. Each tree can be cut into *L* different log types, with each log type having a particular monetary value.

This problem was classified in Climent et al. (2016) as $*/V/D/R$ (see Dyckoff’s typology Dyckhoff (1990)), where $*$ means any dimensionality, *V* means that the total amount of items in stock (i.e. the total number of trees) is sufficient to accommodate all the demanded products (hence, only some of the stock will be cut), *D* means that all large pieces (stock items) are different (in terms of shape) and *R* indicates many products of few different types are demanded. The feature *V* (any demand can be fulfilled) requires that the stock items to be cut need to first be selected. Using the more recent typology presented in Wäscher et al. (2007), the problem can be classified as a variant of the MSSCSP in which there is a heterogeneous assortment of large pieces.

In practice, a semi-autonomous harvesting machine (e.g. Komatsu forest machines) cuts a tree into logs in order to maximise its total value using an algorithm ${\mathcal {A}}$, which is typically based on a Dynamic Programming (DP) algorithm. DP is an approach that enables the solution of complex problems by dividing them into a collection of simpler sub-problems (see Anderson et al. 2015; Bellman and Dreyfus 2015 for example). The sub-problems must be sequential and independent, and the problem of cutting a tree stem satisfies these properties since it is recursive (maximise by cutting the first product and then maximising the cutting of the remainder). Let *L* be the length of a section of a tree stem measured from the base of the stem, and $L_{max}$ be the total usable length of the stem. If $y_k$ is the length of a short log of type *k* cut at a distance $L - y_k$ from the base of the stem, and $(r(y_k, L)$ is the associated product value, this recursive relationship can be represented as

6 $$\begin{aligned} f(L) = \text{ maximum}_{k, y_k \in Y(L)} (r(y_k, L) + f(L-y_k)) \end{aligned}$$

where $f(0) = 0$ and $0 \le L \le L_{max}$ (Eng et al. 1986).

The semi-autonomous nature of the harvesting machines is due to their construction, as they are hard-coded to produce log types with the highest possible monetary value wherever possible. Thus, we only have indirect control over the cutting of trees via a set of *L* continuous variables called a weight vector $\mathbf {w} \in \mathbb {N_+}^{L}$. Each $w_l$ represents the weight (usually the price in €$/m^3$) associated with product type *l*.

Blocks are sold wholesale, i.e. either a block’s trees are completely cut or none of its trees are cut. For each block *q*, a set of product types ${\mathcal {L}}$, a vector of tree dimensions $\mathbf {\sigma }_{qr}$ in the block, and a weight vector $\mathbf {w}_q$ is passed to ${\mathcal {A}}$. The result is a product vector $\mathbf {p}_q \in \mathbb {N_+}^{L}$ showing the total amount of each log type obtained from the block whose trees are cut, denoted by $\mathbf {p}_q = \langle a_1, \dots , a_{L} \rangle$ where $a_l$ represents the volume in $m^3$ of units of log type $l, \,(l=1 \ldots L)$ obtained. Consequently, ${\mathcal {A}}$ can be represented by the following mapping function for block *q*:

7 $$\begin{aligned} {\mathcal {A}}({\mathcal {L}},\langle \mathbf {\sigma }_{q1}, \dots , \mathbf {\sigma }_{qR}\rangle , \mathbf {w}_q) \rightarrow \mathbf {p}_q \end{aligned}$$

Solving ${\mathcal {A}}$ with fixed $\mathbf {w}_q$ assigns values to the $\mathbf {p}_q$ variables, and the value obtained from the piece is $\mathbf {w}_q \cdot \mathbf {p}_q$. In some applications actual market prices are used as the weights. For this problem however, the $\mathbf {w}_q$ are manipulated to obtain the desired product yields. This is the only control we have over how tree stems are cut due to the hard-coding of the harvesting machines.

Given a demand vector $\mathbf {D}$ denoting the desired yield $D_{l}$ for each product type $l \in {\mathcal {L}}$, the problem is to determine which blocks should be selected for harvesting, as well as the weight vector to use for each such block, in order to meet the demands while minimising the total value of the harvested blocks to conserve natural resources. There are no restrictions on which subsets of blocks can be chosen, and the trees in a block are either all cut, or none of them are.

There are several approaches that aim to achieve the desired yield $D_{l}$ in the literature. The authors in Kivinen (2004) and (Kivinen 2006) use genetic algorithms to try to improve the fit between the yields obtained by the harvester and the demand with varying levels of success. In Divvela and Sinha (2012), the authors use a price-weighted apportionment degree (AD) index to try to improve the fit between output and demand. This approach still prioritises logs with higher value and may not fulfil demand, leading to overproduction of unwanted logs and consequently waste. The paper (Malinen and Palander 2004) uses flexible variations on the AD to improve the fit between demand and supply; however their approach is not guaranteed to be optimal. In Marshall et al. (2006), the authors provide three mathematical models for bucking to order using a small set of market prices, targeting certain cutting patterns, and using the AD index, respectively. The paper (Kivinen et al. 2005) compares four different measures to determine the similarity between the demand and output log distributions. None of the four are shown to be superior, even though they can be used in the industry to some extent. The authors in Dems et al. (2015, 2017) use the priority list approach where higher value log types are prioritised. This approach also only considers a few cutting patterns which are assumed to be sufficient, although this is not always the case. The paper (Sessions et al. 1989) adjusts the price iteratively, but using only a small set of prices. In Duffner (1980), the authors also vary price but how they do this is not stated.

The analytics-based heuristic decomposition approach used in this paper is a good fit for this problem since a much larger number of prices can be evaluated, thus creating a good approximation of the distribution relating the prices to the products. Also, separating the harvester operation (${\mathcal {A}}$) from the rest of the linear program using bilevel formulation allows for the more efficient solution of problems with a large number of blocks. Additionally, the use of analytics approaches presents a new way of solving a real-world bilevel problem.

### Bilevel reformulation

The above problem can be naturally modelled as a multiple-follower bilevel optimisation problem. This reformulation of the forest harvesting problem as a bilevel problem is novel, and is one of the contributions of this paper. Here, the leader’s objective is to select a set of blocks to harvest to fulfil demand, while each follower *q* seeks to harvest its block to get the optimal product vector $\mathbf {p}_q$, given a price input $\mathbf {w}_q$.

Define binary variables $h_q=1$ if block *q* cuts its raw stock of trees, and product vectors of integers $\mathbf {p}_{qr}$ to describe the product yields from raw *r* in block *q*’s stock. $V_q$ is the monetary value of block *q* estimated by the forest providers. $\mathbf {\sigma }_{qr}$ are the dimensions of an uncut tree stem *r* in block *q*. The problem is thus:

8 $$\begin{aligned} \begin{array}{r@{}l@{}l} \min _{h_1 \ldots h_Q, \mathbf {w}_1 \ldots \mathbf {w}_Q} &{} \sum _{q=1}^Q V_q h_q &{}\\ \text{ s.t. } &{} &{} \\ &{} \sum _{q=1}^Q \sum _{r=1}^R h_q \mathbf {p}_{qr} \ge \mathbf {D}\\ &{} \\ &{} \text{ where } \text{ each } \mathbf {p}_{qr} \, (q = 1, \ldots , Q) \text{ solves: } \\ &{} \mathbf {p}_{qr} \in \text{ argmax}_{\mathbf {p}_{qr}} {\mathcal {A}}({\mathcal {L}},\mathbf {\sigma }_{qr},\mathbf {w}_q) &{} r=1, \ldots , R\\ &{} \text{ s.t. } \\ &{} h_q \in \{0,1\} &{} q = 1, \ldots , Q \\ &{} \mathbf {p}_{qr} \in {\mathbb {N}}^{L} &{} q = 1, \ldots , Q, \forall r\\ &{} \mathbf {w}_q \in [0,1]^L &{} q = 1, \ldots , Q \end{array} \end{aligned}$$

This is a nonlinear, mixed-integer, bilevel optimisation problem with multiple followers which we call the *Bilevel Cutting Stock Problem with Multiple stock sizes* (BCSPM). It is also large: there might be hundreds of blocks and hundreds of (sampled) trees per block, hence tens of thousands or more follower problems (since ${\mathcal {A}}$ is evaluated for each *r*), as well as a large number of product types. It cannot be solved by classical bilevel methods but it could be tackled by evolutionary methods. Metaheuristic approaches (popularly used in industry) such as (Murphy et al. 2004) and (Dueck and Scheuer 1990) have been tried, with very poor results obtained.

The model above does not have the strong independence property because all the follower problems corresponding to a block share the same variables. It can however be transformed so that it does, by grouping each block’s follower problems into a single problem via new vectors of integer variables $\mathbf {p}_q$, which model the total yield from each block:

9 $$\begin{aligned} \begin{array}{r@{}l@{}l} \min _{h_1 \ldots h_Q, \mathbf {w}_1 \ldots \mathbf {w}_Q} &{} \sum _{q=1}^Q V_q h_q &{}\\ \text{ s.t. } &{} &{} \\ &{} \sum _{q=1}^Q \sum _{r=1}^R h_q \mathbf {p}_{qr} \ge \mathbf {D}\\ &{} \\ &{} \text{ where } \text{ each } \mathbf {p}_{q} \, (q = 1, \ldots , Q) \text{ solves: } \\ &{} \mathbf {p}_q \in \sum _{r=1}^R \text{ argmax}_{\mathbf {p}_{qr}} \, {\mathcal {A}}({\mathcal {L}},\mathbf {\sigma }_{qr},\mathbf {w}_{q}) &{}\\ &{} \text{ s.t. } \\ &{} h_q \in \{0,1\} &{} q = 1, \ldots , Q\\ &{} \mathbf {p}_q \in {\mathbb {N}}^{L} &{} q = 1, \ldots , Q\\ &{} \mathbf {w}_q \in [0,1]^L &{} q = 1, \ldots , Q \end{array} \end{aligned}$$

Now the followers are strongly independent: each uses a unique set of variables $\mathbf {w}_q,\mathbf {p}_{q}$ and none of the follower variables are mutually constrained. The decomposition method detailed in Sect. 4 can now be applied.

For each $\mathbf {w}_q$ a number of feasible solutions ($\mathbf {W}_{sq}$) are generated. Each follower problem is then solved for the $\mathbf {W}_{sq}$ using the cutting simulator ${\mathcal {A}}$, to get corresponding $\mathbf {P}_{sq}$. Next, *k*-medoids clustering is applied for each follower *q*, resulting in the selection of a diverse set of assignments $\mathbf {P}_{kq}$, together with the corresponding $\mathbf {W}_{kq}$. The problem can now be formulated as an ILP:

10 $$\begin{aligned} \begin{array}{r@{}l@{}l} \min _{h_1 \ldots h_Q, \mathbf {w}_1 \ldots \mathbf {w}_Q} &{} \sum _{q=1}^Q V_q h_q &{}\\ \text{ s.t. } &{} &{} \\ &{} \sum _{q=1}^Q \sum _{k=1}^K P_{kql} x_{qk} \ge D_1 &{} l = 1, \ldots , L\\ &{} \sum _{k=1}^K x_{qk}=h_q &{} q = 1, \ldots , Q\\ &{} h_q \in \{0,1\} &{} q = 1, \ldots , Q \\ &{} x_{qk} \in \{0,1\} &{} q = 1, \ldots , Q, \, k = 1, \ldots , K \\ \end{array} \end{aligned}$$

where $h_q=1$ indicates that all block *q*’s trees are cut, and $x_{qk}=1$ indicates that they are cut using weights with index *k*. If block *q* is not selected then $h_q=0$ which forces $x_{qk}=0$ for $k=1 \ldots K$.

### Evaluation of the bilevel cutting stock problem

**Fig. 7:**
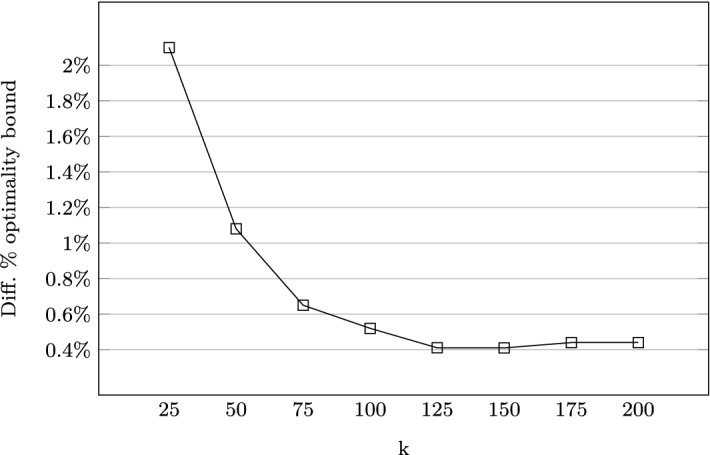
Percentage optimality difference (Prestwich et al. 2015)

To empirically study the performance of our approach, real data from an industrial partner was used. A smaller evaluation using 8 blocks (Q = 8) with each block’s trees partitioned into a maximum of 4 different types of products was done in Prestwich et al. (2015). Results showed that the approach came to within $0.4\%$ of optimality bound (see Fig. 7). It was also seen that the total clustering time increased exponentially with *k*.

A larger evaluation was carried out on a different forest with 263 blocks ($Q = 263$) and the stock partitioned into a maximum of 11 product types. The total volume of raw material was $6149.781\,m^3$, with the data obtained from the industrial partner. 1000 random weight vectors $\mathbf {W}_{sq}$, ($S=1000$) were generated for each block, giving a total of 263,000 cutting pattern vectors $\mathbf {P}_{sq}$. The total time for generating these was approximately 18 h and 17 min. The total clustering time for all blocks with $k=125$ was 31 h. 12 different instances of random demands were solved. In 6 of these instances, the demand for product types was in the range $[0,300]\,m^3$ (low demand), while the remaining 6 had demands in the range $[300,600]\,m^3$ (high demand). ILP solution times were nominal, taking less than 5 s for all instances evaluated.

Due to the high clustering times, self-organising maps (SOM) were used an alternative to *k*-medoids. The SOM experiments were done using the Java Kohonen Neural Network Library (JKNNL) Rybarski and Habdank-Wojewódzki (2006). For the small problem ($Q=8$), the SOM was trained on the 80,000 cutting pattern vectors $\mathbf {P}_{sq}$, using a varying number of neurons $N \in \{ 25, 50, 75, 100, 125, 150, 175, 200\}$ arranged in a grid topology.

**Fig. 8:**
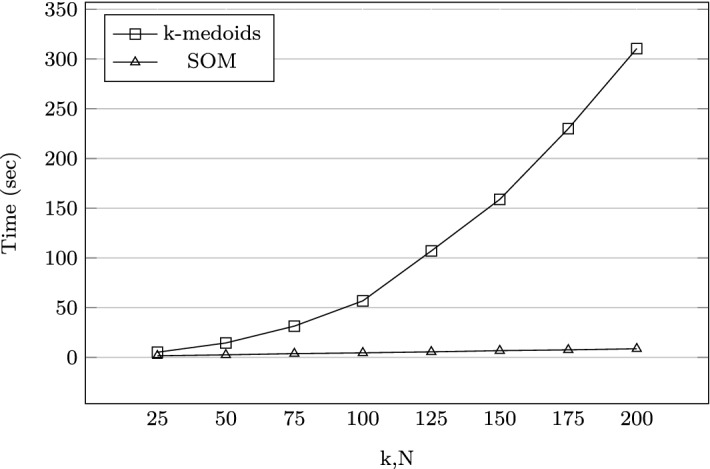
Scalability of clustering approaches for one block

A chart comparing the increase in clustering times for both *k*-medoids and SOM approaches is shown in Fig. 8.

**Fig. 9:**
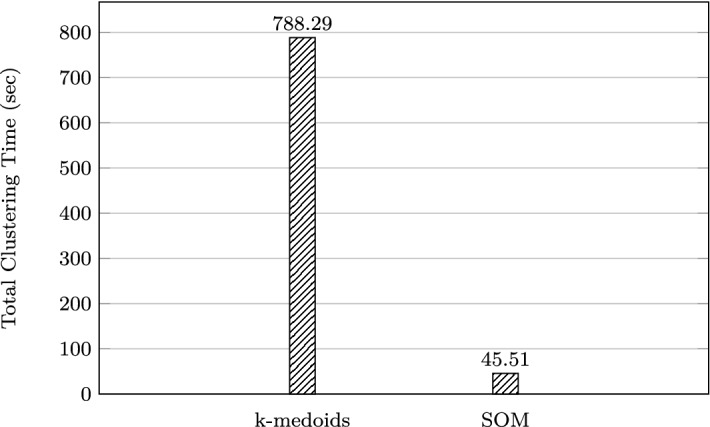
Comparing clustering times for a small problem instance ($Q = 8$),$k = N = 125$

**Fig. 10:**
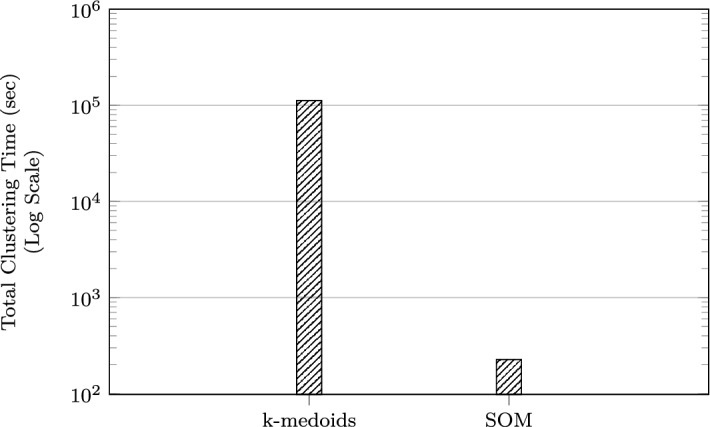
Comparing clustering times for a large problem instance ($Q = 263$),$k = N = 125$

In terms of scalability, it can be seen in Fig. 8 that using the SOM approach offers a significant improvement in data reduction time, which makes it more useful than *k*-medoids for large problems. In Fig. 9, when $k=N=125$ for $Q=8$, the total clustering times are 788.29 and 45.51 s for the *k*-medoids and SOM, respectively. When $Q = 263$, the difference in clustering and map training times is even more striking and is better visualised using a logarithmic scale (Fig. 10). Here, the total times taken are 111898.35 s (31 days) and 226.72 s (4 min) for the *k*-medoids and SOM, respectively.

## Conclusions

In this paper, a novel analytics-based heuristic decomposition approach for new class of bilevel multiple-follower problems is presented. Our approach can be used to solve large-scale multiple-follower bilevel problems more efficiently than standard approaches, as complete approaches are not practicable due to excessive computational times.

Two numerical examples were solved using this approach, and the results compared to those obtained by using evolutionary algorithms (which is a standard approach for large-scale bilevel problems). For the first example, a toy problem is solved for demonstration purposes to within 0.09% of the optimal. This shows that even for small-scale problems, the analytics approach is competitive as it is able to cover the space of the follower problems adequately.

The second example was an arbitrarily large problem evaluated for up to a thousand followers. The results were compared with those from two evolutionary approaches and it was seen that as the size of the problem increased, the heuristic decomposition approach produced significantly better results than the standard approaches. This shows that the decomposition approach is much more scalable as the number of followers increases, in terms of both runtime and solution quality.

Besides, a large-scale MSSCSP with applications in the forestry industry was evaluated. This problem was reformulated as a large-scale multiple-follower bilevel problem and solved using the heuristic decomposition method. Evaluation on a small-scale problem showed that up to a point, increasing the number of clusters got the solution to about $0.4\%$ from the optimal. To reduce the clustering time required for large problems, self-organising maps were used as an alternative to *k*-medoids clustering with significant speed-up seen.

A possible future line of work would be the extension of the presented analytics-based heuristic decomposition method to non-independent followers. Furthermore, our approach could be applied to other complex industrial applications that can be modelled as multi-follower bilevel problems

## Footnotes

1 These are a way of expressing if-else relationships among variables (IBM 2017).
